# Anatomy, aetiology, clinical presentation, classification, diagnosis, and non-surgical management of triangular fibrocartilage complex tears: a narrative review

**DOI:** 10.3389/fspor.2026.1695616

**Published:** 2026-10-01

**Authors:** Peng Liu, Tianyu Zhou

**Affiliations:** 1 Faculty of Health Sciences and Sports, Macao Polytechnic University, Macao, China; 2 Hunan Institute of Sports Science, Changsha, Hunan, China; 3 School of Exercise and Health, Shanghai University of Sport, Shanghai, China; 4 MSk Lab, Department of Surgery & Cancer, Imperial College London, London, United Kingdom

**Keywords:** classification, diagnosis, non-surgical management, triangular fibrocartilage complex, wrist injuries

## Abstract

**Background:**

Triangular fibrocartilage complex (TFCC) injuries are a common source of ulnar-sided wrist pain and functional impairment, particularly in athletes and individuals engaged in upper-limb-dominant or wrist-loading activities. Despite their prevalence, standardised, non-surgical treatment pathways remain underdeveloped.

**Hypothesis/purpose:**

This narrative review aims to critically synthesise and discuss current evidence on the non-surgical management of TFCC injuries in physically active adults.

**Study design:**

Narrative review.

**Methods:**

A structured literature search was performed in PubMed, Scopus, Web of Science, and the Cochrane Library for English-language studies published from January 2015 to May 2025. The identified studies focusing on the non-surgical management of TFCC injuries were screened and included.

**Results:**

Current evidence suggests a range of non-surgical interventions for pain reduction and functional improvement in TFCC injuries across different clinical presentations, including rest, splinting, physical therapy, NSAIDs, corticosteroid injections, and progressive functional rehabilitation. Adjunctive modalities such as extracorporeal shock wave therapy and platelet-rich plasma injections also show potential benefits. However, important gaps persist in TFCC classification–specific treatment recommendations, the standardisation of rehabilitation protocols, and the integration of emerging therapies.

**Conclusion:**

This review synthesises current evidence on the non-surgical management of TFCC injuries. Recognising differences in injury mechanism, particularly between acute trauma-related and repetitive overuse-related degenerative presentations, it proposes a structured, phase-based rehabilitation framework for TFCC lesions with an intact distal radioulnar joint. The framework emphasises presentation-specific early management, progressive tissue loading, and functional reintegration.

**Clinical relevance:**

This review provides clinicians with a conceptual, non-surgical framework to guide the management of TFCC injuries, aiming to support pain reduction and functional recovery in physically active adults.

**What is known about this subject:**

While surgical options for TFCC injuries are well established, standardised non-surgical pathways remain underdeveloped and diagnosis can be challenging due to overlapping presentations.

**What this study adds to existing knowledge:**

This review summarises current evidence on non-surgical TFCC management, including conventional and emerging therapies, and outlines a stage-based approach derived from available literature and clinical reasoning to support individualised non-surgical care.

## Introduction

The triangular fibrocartilage complex (TFCC), situated on the ulnar side of the wrist, plays a key role in stabilising the distal radioulnar and ulnocarpal joints and distributing load during wrist motion (1). Due to its complex anatomy and the mechanical demands placed upon it, the TFCC is prone to injury (2). These injuries typically present as non-specific ulnar-sided wrist pain and functional impairment, particularly in populations engaged in repetitive forearm rotation and axial loading, such as gymnasts, racquet-sport athletes (e.g., tennis players), baseball players, golfers, and weightlifters, as well as manual labourers (3). TFCC pathology can result from both degenerative and traumatic mechanisms (4). Degenerative changes are common in older adults, affecting up to 49% of individuals aged 70 years and older (5). Epidemiological studies have also reported a slightly higher prevalence of degenerative TFCC changes in women, possibly related to age-associated connective tissue degeneration and hormonal factors (6). In contrast, traumatic TFCC tears are predominantly observed in athletes, accounting for approximately 3%–9% of all hand and wrist injuries (7).

Despite their prevalence, timely and accurate diagnosis remains challenging. This difficulty arises because TFCC pathology frequently overlaps in presentation with other ulnar-sided wrist disorders, including ulnar impaction syndrome, extensor carpi ulnaris (ECU) tendinopathy, and lunotriquetral ligament injury (8). As a result, patients may report similar clinical features—such as mechanical clicking, reduced grip strength, and pain provoked by forearm rotation or ulnar deviation—making clear differentiation difficult in routine practice (8).

These challenges are further compounded by the absence of standardised diagnostic criteria and universally accepted classification systems, particularly in primary care or early clinical evaluations (2, 9). Such ambiguity not only delays diagnosis but also complicates treatment decision-making, especially in distinguishing patients who may benefit from conservative management from those requiring surgical intervention (10). In addition, there is currently no structured, progressive rehabilitation protocol specifically tailored for the non-surgical management of TFCC injuries.

Several recent high-quality reviews have advanced understanding of the diagnostic imaging and surgical management of TFCC injuries (2, 11, 12). Specifically, Treiser et al. conducted a meta-analysis comparing the diagnostic accuracy of MRI and MR arthrography for TFCC tears (2), while Demino et al. synthesised clinical outcomes associated with different surgical repair techniques (11). More recent publications have further explored evolving concepts and trends in surgical management (12). However, these advances have been largely confined to imaging strategies and operative interventions, with conservative management often addressed only briefly and without detailed guidance on treatment progression or modality-specific evidence. In routine clinical practice, particularly in early-stage presentations or non-specialist settings, this lack of structured, evidence-informed non-surgical pathways may contribute to variability in management decisions. To address this gap, this review aims to synthesise the current evidence on non-surgical interventions for TFCC injuries and to propose a structured, phase-based rehabilitation framework informed by existing clinical data.

## Main body

### Search strategy

A structured literature search was conducted to identify all relevant publications pertaining to the non-surgical management of TFCC injuries. In consultation with a specialist medical librarian, we searched four electronic databases: PubMed, Scopus, Web of Science, and the Cochrane Library from January 2015 until May 2025. This time limit was deliberately applied to ensure a focus on contemporary, high-quality evidence, reflecting the significant advancements in TFCC pathophysiology and the evolution of diagnostic and classification systems since 2015. Seminal publications published before this period were also consulted and cited where necessary to ensure that key historical concepts were appropriately represented. The core search terms included: (“triangular fibrocartilage complex” OR TFCC) AND (conservative OR “non-operative” OR “non-surgical” OR management OR rehabilitation OR physiotherapy OR injection OR “platelet-rich plasma” OR PRP OR “extracorporeal shockwave therapy” OR ESWT).

### Study selection and eligibility

The initial search results were screened against pre-defined eligibility criteria. Studies were included if they: (1) involved human adults with TFCC injuries; (2) focused on anatomy, classification, diagnosis, or any form of non-surgical management; and (3) were original research (e.g., randomised controlled trials, cohort studies), systematic reviews, or high-quality narrative reviews published in English. Exclusion criteria were: (1) studies exclusively on surgical techniques; (2) small case reports (*n* < 5); and (3) conference abstracts and editorials.

The study selection process was conducted by two independent reviewers (PL and TZ). After duplicate removal, records were screened by title and abstract, followed by a full-text assessment of potentially eligible articles. Any discrepancies were resolved through consensus. Given the narrative nature of this review, a formal risk-of-bias assessment or meta-analysis was not performed. The synthesis of evidence is therefore descriptive and aims to provide a comprehensive overview rather than a quantitative summary.

### Clinically relevant anatomy

TFCC extends from the medial margin of the distal radius to the base of the ulnar styloid process and comprises several key structures: the triangular fibrocartilage disc proper (TFC), the dorsal and volar distal radioulnar ligaments, the meniscal homologue, the ulnocarpal collateral ligament, the ulnotriquetral and ulnolunate ligaments, and the ECU tendon subsheath (13) (Figure 1). Vascular supply is limited to approximately 20% of the TFCC's peripheral region, while the central 80% consists predominantly of avascular fibrocartilaginous tissue (14) (Figure 2). This distribution has important clinical implications, as peripheral tears within the vascular zone tend to heal better and respond more favourably to conservative treatments such as immobilisation or biologics, whereas central degenerative lesions are less likely to benefit from non-surgical approaches.

**Figure 1:**
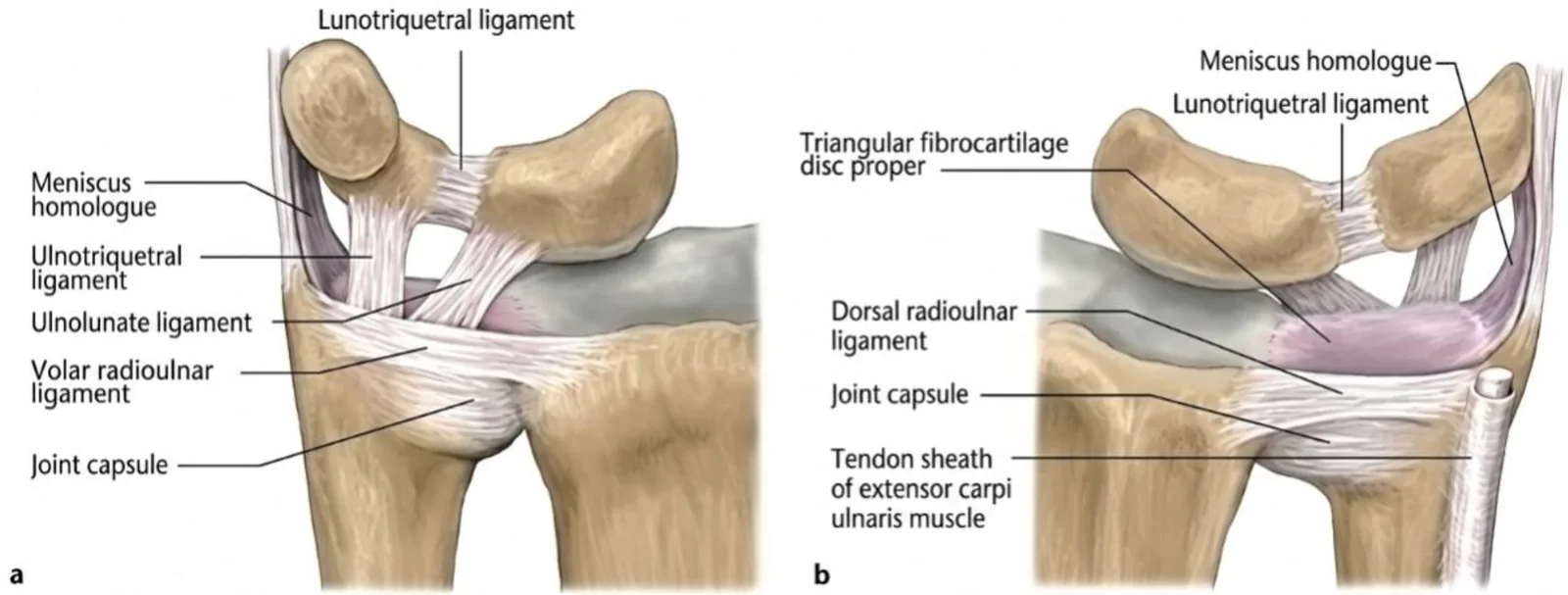
Illustration of TFCC anatomy: **(a)** palmar view; **(b)** dorsal view. (Adapted from: Fife Virtual Hand Clinic. Injuries to the Triangular Fibrocartilaginous Complex (TFCC).

**Figure 2:**
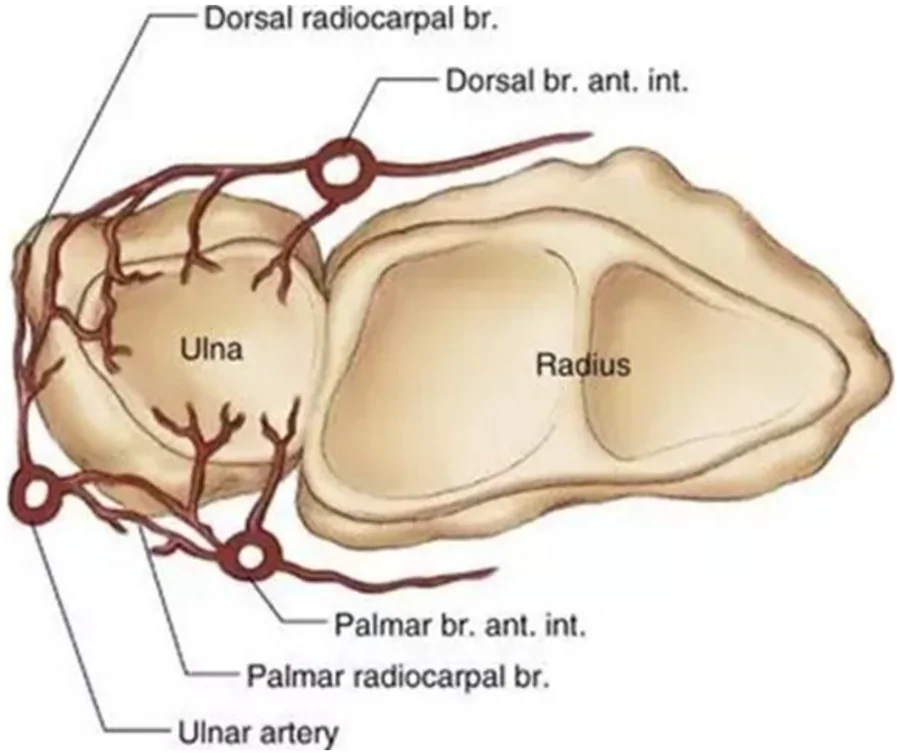
Illustration of the blood supply to the TFCC. (Adapted from Thiru-Pathi RG, Ferlic DC, Clayton ML, et al. Arterial anatomy of the triangular fibrocartilage of the wrist and its surgical significance. J Hand Surg. 1986;11A:261.).

In addition to its vascular characteristics, the TFCC demonstrates region-specific neural innervation. Histological studies have identified free nerve endings and mechanoreceptors—particularly Ruffini endings and Pacinian corpuscles—within the peripheral portions of the TFCC, especially in the dorsal and ulnar regions. In contrast, the central fibrocartilaginous disc is largely aneural (15). This distribution is clinically relevant, as peripheral tears are more likely to produce pain due to sensory innervation, whereas central degenerative lesions may be less symptomatic despite structural damage (16).

The largest component, the fibrocartilaginous disc, extends from the articular cartilage at the sigmoid notch of the radius to the ulnar styloid and the ulnar fovea (17). At its radial attachment, the TFCC forms a dense fibrous band that divides into proximal and distal laminae separated by fibrovascular tissue known as the ligamentum subcruentum. The proximal lamina inserts into the ulnar fovea, while the distal lamina attaches to the distal ulnar styloid; together, these structures form what is referred to as the triangular ligament (18).

### Aetiology

TFCC injuries arise from mechanical loading through two primary mechanisms: overuse-related degenerative changes and acute traumatic tears (4). Overuse-related pathology typically results from repetitive compressive loading, particularly during sustained or repeated ulnar deviation. This loading increases ulnocarpal contact stress and contributes to progressive degenerative changes over time (19). In contrast, acute traumatic tears are generally caused by high-load shear and torsional forces, such as those generated during forceful forearm rotation or a fall onto an outstretched hand (20, 21). In sporting contexts, cumulative repetitive ulnar deviation—common in racket and bat sports—is primarily associated with degenerative TFCC changes, whereas sudden high-energy rotational or axial loads, such as a gymnast landing on an outstretched hand, a baseball player sliding into base, or a weightlifter catching a heavy bar, are more likely to produce acute traumatic tears (22).

Anatomical and biomechanical factors further modulate these mechanical stresses. Specifically, ulnar variance—the relative length of the distal ulna compared with the distal radius—affects load transmission across the ulnocarpal joint and is a well-established risk factor for TFCC injury (23). Ulnar variance dynamically decreases with supination and increases with pronation, and even subtle changes in ulnar length can substantially alter ulnocarpal load distribution (24). These dynamic changes also influence the mechanics of surrounding stabilising structures, particularly the ECU subsheath, which functions as a dynamic pulley system within the TFCC. Alterations in ECU kinematics can impose abnormal stresses on the TFCC, thereby exacerbating injury risk (25).

From a clinical perspective, the influence of ulnar variance and related biomechanical factors on outcomes following TFCC injury remains controversial. While some studies associate positive ulnar variance with greater pain and degenerative progression (26, 27), others report no clear relationship with functional outcomes after conservative management (28). These discrepancies likely reflect differences in injury mechanisms, tissue healing capacity, and patient activity demands. Notably, athletes are exposed to higher mechanical loads and accelerated return-to-sport pressures, which may predispose them to poorer outcomes or earlier surgical intervention compared with the general population (7, 22).

Beyond biomechanical considerations, recovery trajectories are also influenced by personal and environmental factors. Patients' professional demands, social roles, and personal expectations can affect adherence to rehabilitation, tolerance of residual pain, and perceived functional recovery (29). Incorporating a biopsychosocial perspective may help clinicians individualise rehabilitation plans and better predict functional outcomes, highlighting the interaction between mechanical, psychological, and social determinants of TFCC recovery (29). Together, these findings suggest that aetiological, biomechanical, and psychosocial factors collectively shape injury risk, recovery patterns, and treatment pathways.

### Clinical presentation

Patients with TFCC injuries commonly present with ulnar-sided wrist pain, which is often activity-related and exacerbated by wrist rotation, such as turning a doorknob or unscrewing a jar (19). Other frequent features include decreased grip strength and a subjective sense of distal radioulnar joint instability, often described as weakness, giving-way, or a feeling of looseness or lack of control during functional activities such as pushing open a door, weight-bearing through the hand, or forearm rotation (8). Mechanical symptoms such as clicking, catching, or locking during wrist movement may also be reported (1, 8). Swelling or bruising is more typical of acute traumatic injuries, whereas overuse-related or degenerative lesions tend to be associated with subtler, insidious pain (30). Symptom patterns and severity vary widely among patients, underscoring the importance of careful clinical assessment of functional limitations and activity-specific symptom triggers. Some studies suggest gender-related differences: traumatic TFCC injuries are more commonly observed in male athletes, whereas degenerative TFCC lesions tend to be more prevalent in older female populations (1, 5).

### Classiﬁcation

The Palmer classification remains the most widely recognised system for categorising TFCC tears, distinguishing traumatic (Type 1) from degenerative (Type 2) lesions (Table 1) (1). Type 1 traumatic lesions result from acute trauma and include isolated central perforations of the fibrocartilage disc (1A), peripheral ulnar-sided tears of the TFCC (1B), distal disruption of the TFCC involving the ulnocarpal ligaments (1C), and radial avulsions affecting the radioulnar ligament attachments (1D) (17). Central tears (1A) occur in the relatively avascular zone of the disc and therefore have limited healing potential, whereas peripheral tears (1B–1D) benefit from better vascular supply and are generally more amenable to repair (14). In contrast, Type 2 degenerative lesions arise from chronic ulnar-sided loading, often associated with ulnar impaction, and represent a progressive spectrum of degeneration involving the TFCC and surrounding structures (31). They are further subclassified from 2A to 2E according to severity: 2A represents degenerative changes of the triangular fibrocartilage disc without perforation; 2B includes these degenerative changes with additional chondromalacia of the adjacent articular cartilage; 2C represents a full-thickness perforation of the triangular fibrocartilage disc; 2D includes the features of 2C with associated lunotriquetral ligament tear or cartilage abnormalities of the ulnar head and lunate; and 2E represents advanced disease characterised by ulnocarpal arthritis (1).

**Table 1:** Palmer classification of TFCC injuries.

Type		Description	Illustration
Palmer Class 1: Traumatic	1A	Isolated central disk perforation	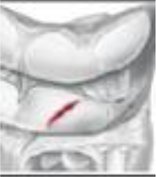
	1B	Peripheral ulnar-sided tear of TFCC (with or without ulnar styloid fracture)	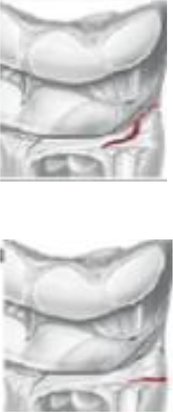
	1C	Distal disruption of the TFCC (with or without sigmoid notch fracture)	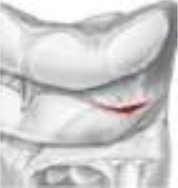
	1D	Injury to the radial attachment of the triangular fibrocartilage disc	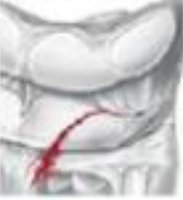
Palmer Class 2: Degenerative	2A	Degenerative changes of the triangular fibrocartilage disc without evidence of perforation	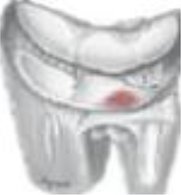
	2B	Grade 2A with the additional presence of chondromalacia of the hyaline cartilage on the articular surface	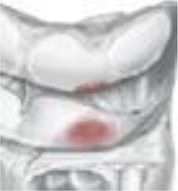
	2C	Full-thickness perforation of the triangular fibrocartilage disc	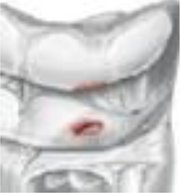
	2D	Any features in 2A through 2C plus lunotriquetral ligament tear or cartilage abnormalities of the ulnar head and the lunate.	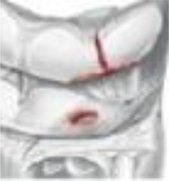
	2E	Grade 2D with the additional presence of ulnocarpal arthritis	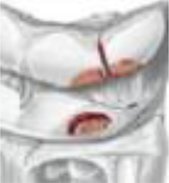

Adapted from Palmer (1) and Spies (36). Minor wording modifications were made for clarity and consistency with the terminology used in this review.

While Palmer's system is widely used, it does not fully capture features critical for guiding treatment, such as tear location (volar vs dorsal), thickness, configuration, distal radioulnar joint (DRUJ) stability, or combined injuries (32). To address these gaps, the Atzei classification refines the evaluation of peripheral tears by assessing foveal and styloid attachment integrity and DRUJ stability (9). For example, Palmer 1B lesions can be further sub-classified: stable Atzei type 1 tears (partial peripheral involvement with preserved DRUJ stability) are typically managed non-surgically, whereas unstable Atzei type 2 or 3 tears (complete foveal disruption with DRUJ instability) usually require surgical repair to restore anatomy and function (33). Evidence supports this approach, as repair of peripheral lesions improves functional outcomes compared with debridement alone, particularly in tears with structural instability (34, 35).

### Diagnostic procedures

To facilitate clinical decision-making, the diagnostic evaluation of suspected TFCC injuries can be conceptualised as a structured diagnostic pathway integrating clinical history, physical examination, imaging modalities, and differential diagnosis (Figure 3). This stepwise approach helps clinicians determine when advanced imaging or surgical evaluation is required.

**Figure 3:**
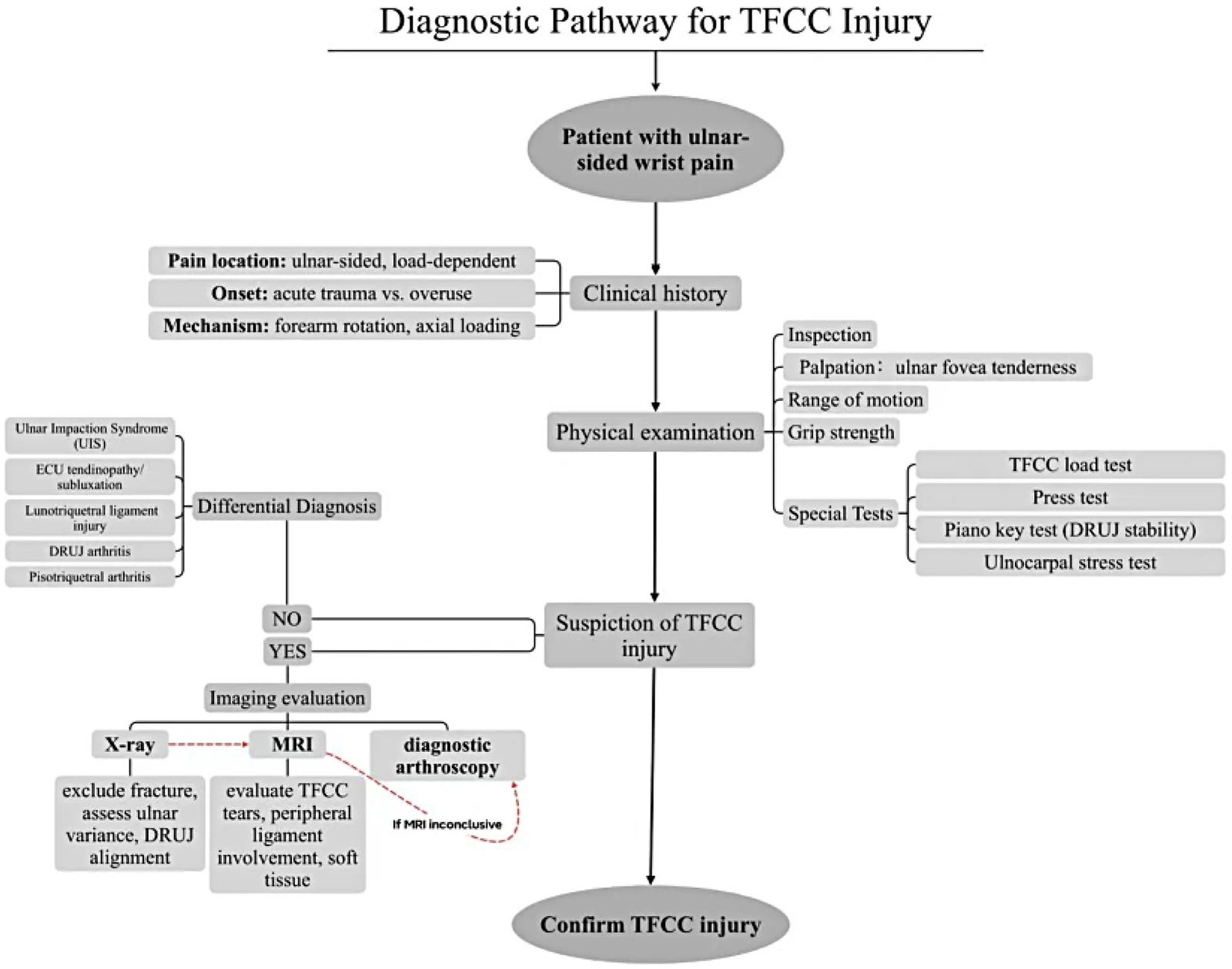
Clinical diagnostic pathway for suspected TFCC injury.

**Figure 4:**
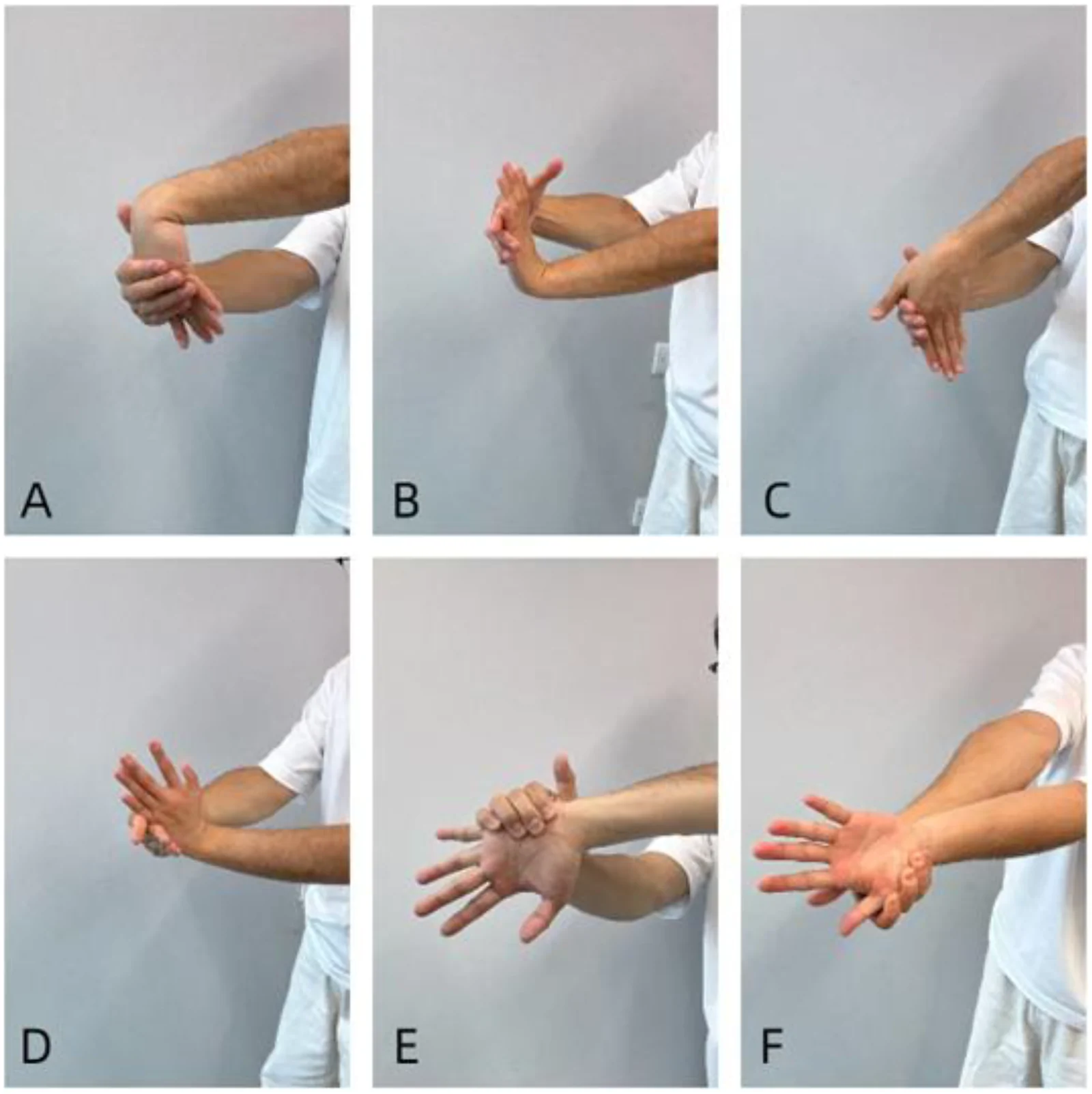
General mobility exercises: **(A)** flexion; **(B)** extension; **(C)** ulnar deviation; **(D)** radial deviation; **(E)** pronation; **(F)** supination. Diagram created by the authors.

**Figure 5:**
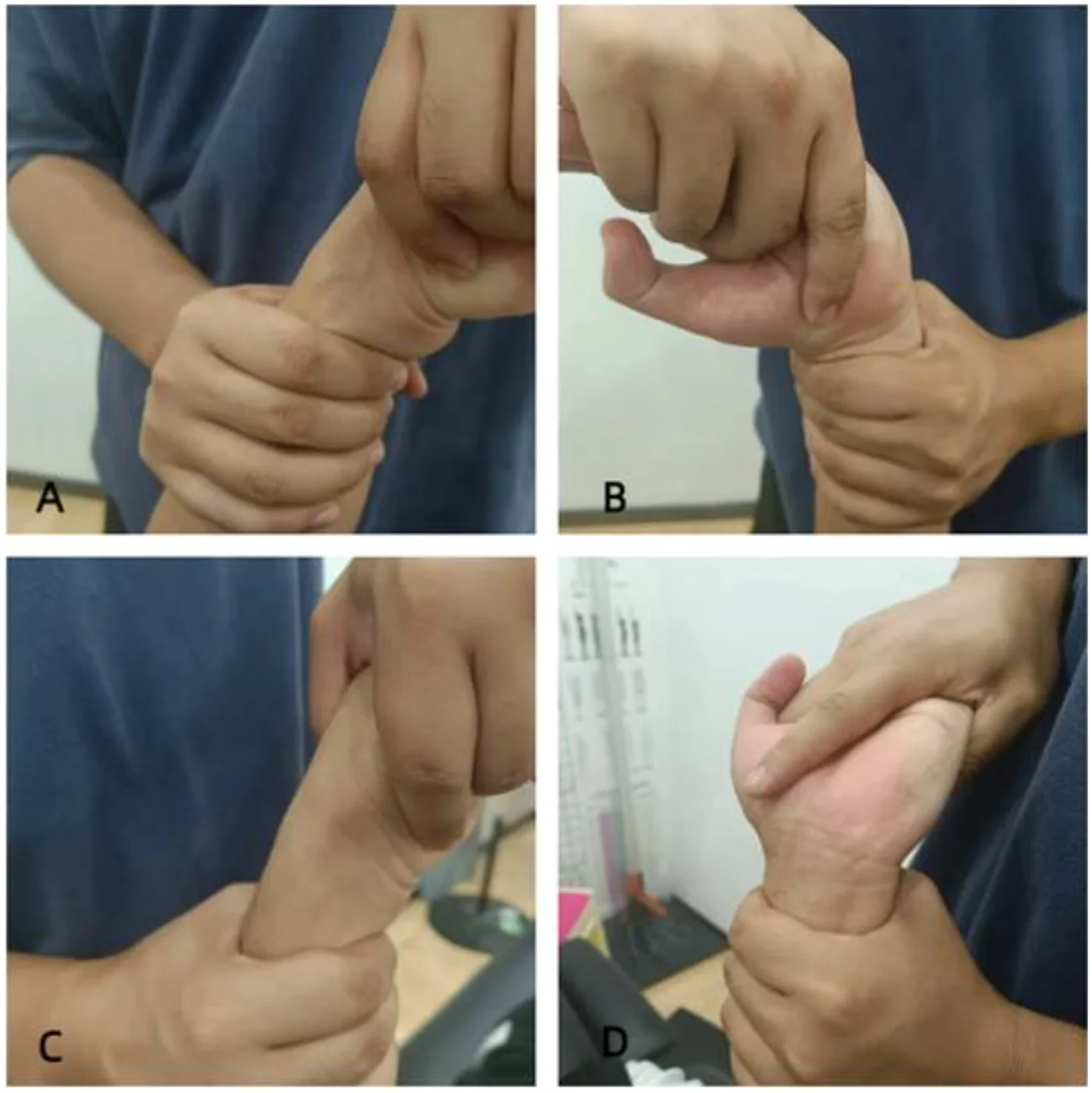
Passive mobilisation techniques of the wrist. **(A)** Radial glide of the proximal carpal row to improve ulnar deviation; **(B)** Ulnar glide to improve radial deviation; **(C)** Volar glide to improve wrist extension; **(D)** Dorsal glide to improve wrist flexion. Glides are performed within a pain-free range and according to patient tolerance. Diagram created by the authors.

**Figure 6:**
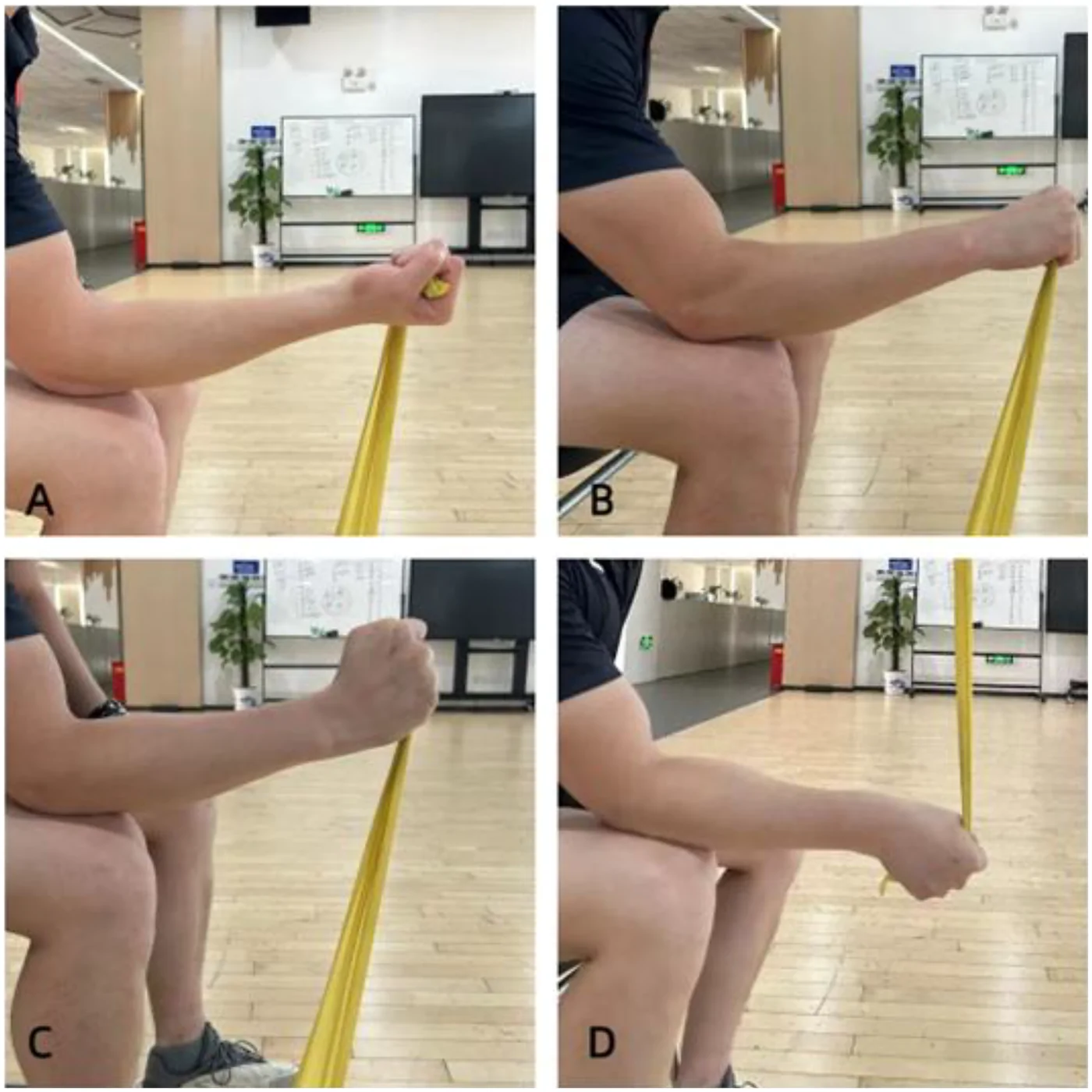
Isometric contraction exercises for wrist rehabilitation. **(A)** Wrist flexion; **(B)** Wrist extension; **(C)** Radial deviation; **(D)** Ulnar deviation. Exercises should be performed within a pain-free range, initially in a neutral wrist position, with gradual progression based on symptom tolerance. Diagram created by the authors.

### Diagnostic imaging

The diagnostic evaluation of suspected TFCC injuries relies on a combination of imaging and surgical modalities. Currently, arthroscopy remains the diagnostic gold standard, providing direct visualisation of the articular disc and associated structures across all TFCC regions (8, 37), and is indicated when non-invasive imaging is inconclusive or when surgical intervention is planned. For non-invasive evaluation, magnetic resonance imaging (MRI) is considered the first-line modality for soft-tissue assessment of the TFCC (38), demonstrating good overall diagnostic performance (sensitivity 92%, specificity 89%, accuracy 91%) (39).

However, these primary modalities present notable limitations. MRI has reduced sensitivity for ulnar-sided or peripheral lesions, including peri-foveal and distal ulnar insertions, and may miss subtle disruptions in the central recess (40). When these limitations are clinically relevant, MR arthrography can improve visualisation of intra-articular structures, though its incremental benefit over conventional MRI is debated due to added invasiveness, cost, and patient discomfort (41). To address these limitations and provide a comprehensive evaluation, several adjunct approaches are integrated into the diagnostic pathway. Initial assessment typically begins with plain radiography, which serves as a highly accessible adjunct to exclude fractures and evaluate ulnar variance. Furthermore, computed tomography (CT) is not routinely used for TFCC assessment but may be considered as an alternative adjunct when MRI is unavailable or contraindicated, recognising its limited soft-tissue contrast (2).

### Special tests

Although imaging plays an important role in TFCC evaluation, physical examination remains the first-line diagnostic approach and is particularly valuable for determining the need for advanced imaging. Prior to performing specific manoeuvres, the initial clinical assessment should include inspection, palpation of the ulnar fovea (the depression between the ulnar styloid and the flexor carpi ulnaris tendon that corresponds to the attachment of the deep radioulnar ligaments) (14), range-of-motion testing, and functional tasks such as resisted forearm rotation and weight-bearing tolerance to establish baseline symptom behaviour. Several clinical tests are commonly used to provoke TFCC-related symptoms:

Ulnar Fovea Sign: Palpation of the ulnar fovea is performed with the patient's wrist in neutral while the examiner applies firm pressure into the soft tissue depression between the ulnar styloid and the flexor carpi ulnaris tendon. Reproduction of focal tenderness compared with the contralateral wrist constitutes a positive finding. Given its high reported diagnostic accuracy (sensitivity ≈ 95.2%, specificity ≈ 86.5%) (42), a positive ulnar fovea sign should raise strong suspicion for a foveal TFCC tear and prompt careful assessment for DRUJ instability, which may necessitate surgical referral.

Ulnocarpal Stress Test: With the patient seated and the forearm in neutral rotation, the examiner stabilises the distal forearm while applying axial compression through the palm with the wrist positioned in ulnar deviation. The manoeuvre can be performed both in static loading and while gently rotating the wrist through pronation and supination. Reproduction of ulnar-sided wrist pain, clicking, or grinding sensations suggests TFCC pathology or ulnocarpal joint irritation (8, 19).

Load Simulation Tests: Several provocative manoeuvres simulate functional loading across the TFCC and may reproduce symptoms. The supination test involves the patient actively supinating the forearm while gripping the edge of a table or similar surface to generate compressive load across the TFCC. Pain localised to the ulnar side of the wrist during this manoeuvre is considered a positive finding (19). Similarly, the ulnar grinding test is performed with the wrist in ulnar deviation under axial compression while the examiner applies rotational movements. This manoeuvre mimics load transmission across the ulnocarpal joint and may reproduce pain, crepitus, or mechanical symptoms consistent with TFCC injury (43).

Piano Key Test (DRUJ Ballottement Test): With the patient's forearm resting on a stable surface, the examiner stabilises the radius and applies downward pressure to the distal ulna. Excessive dorsal–volar translation of the ulna relative to the radius—resembling the depression of a piano key—indicates distal radioulnar joint instability and may suggest a peripheral TFCC tear involving the foveal attachment (3, 44).

Although these clinical tests are widely used in practice, evidence regarding their diagnostic specificity remains limited. Only a small number of studies have reported validated diagnostic accuracy values. Importantly, a positive result is generally defined by reproduction of the patient's characteristic ulnar-sided wrist pain, often accompanied by mechanical symptoms such as clicking, grinding, or subjective instability. Symptoms that are mild, poorly localised, or reproduced similarly on the contralateral wrist are typically considered non-specific and may not indicate clinically meaningful TFCC pathology (45).

### Differential diagnosis

Given the overlap in clinical presentation, careful differentiation of TFCC injuries from other causes of ulnar-sided wrist pain is essential for accurate diagnosis and appropriate management. Physical examination findings can assist in guiding differential diagnosis, although their interpretation is partly operator-dependent and influenced by examiner experience (46).

Ulnar impaction syndrome typically provokes pain during the ulnar impaction test and may produce positive findings during grip-provocative manoeuvres, including the ulnar fovea sign and the ulnar compression test (45). ECU tendinopathy often presents with localised tenderness, snapping, or subluxation over the ECU groove, particularly during resisted wrist extension or ulnar deviation (47). Lunotriquetral ligament injuries may be suggested by mechanical clicking and instability elicited during the lunotriquetral ballottement test (48). DRUJ arthritis should also be considered, as it may cause chronic ulnar-sided wrist pain, restricted DRUJ range of motion, and joint-localised tenderness (49). Unlike TFCC injuries, DRUJ arthritis commonly demonstrates radiographic features such as joint space narrowing, osteophyte formation, and subchondral sclerosis, consistent with a degenerative process (50).

### The role of surgical treatment

The management of TFCC injuries is primarily guided by the biomechanical stability of the DRUJ and the biological healing potential of the injured tissue. In general, non-surgical treatment is prioritised as the first-line approach for clinically stable tears and degenerative lesions, whereas surgical intervention is reserved for unstable traumatic injuries or cases that remain symptomatic despite an adequate trial of conservative rehabilitation (35, 51). Surgical repair is considered the gold standard for acute traumatic TFCC injuries involving foveal avulsion, particularly Palmer type 1B lesions, as these injuries disrupt the primary ligamentous stabilisers of the DRUJ (52). Early surgical repair has been reported to achieve success rates exceeding 95%, restoring joint stability and functional outcomes (53). Surgical intervention may also be considered in elite athletes requiring rapid return to sport or in patients with associated injuries such as Galeazzi fracture–dislocations (12).

Although the Palmer classification remains widely used to categorise TFCC tears, it does not directly assess foveal involvement or DRUJ stability. The Atzei classification therefore provides additional clinical value by further subclassifying peripheral TFCC tears based on the integrity of the foveal attachment and the presence of DRUJ instability, thereby helping guide treatment selection.

Therefore, this review emphasises that conservative treatment is not a universal solution for all TFCC pathology. Instead, it is strictly indicated for: (1) stable tears without DRUJ instability (e.g., Palmer 1A, 1C, 1D); (2) degenerative tears without clinically significant mechanical symptoms (e.g., symptomatic clicking, catching, or load-dependent instability); and (3) patients who are not suitable surgical candidates or who explicitly prefer to avoid surgery (35, 51). Recognising this mechanistic distinction is essential for appropriate patient selection and achieving optimal clinical outcomes.

### Non-surgical treatment

Non-surgical management is the first-line approach for patients with clinically stable TFCC injuries—specifically Palmer Types 1A, 1C, 1D, and stable Type 2 degenerative lesions without DRUJ instability—as well as for those who are not suitable candidates for surgery (8, 19). The primary aims are to reduce pain, protect healing tissues, and restore function while avoiding unnecessary operative intervention. Initial management typically includes activity modification, short-term wrist support to offload the TFCC, and pharmacological symptom control using NSAIDs or, when appropriate, targeted corticosteroid injections (54). Following this early phase, conservative care is centred on physiotherapy-led rehabilitation to address impairments in wrist motion, strength, neuromuscular control, and functional use (55). A supervised trial of non-surgical management is typically recommended for a duration of 3–6 months (35, 56). While a rigid temporal cutoff is lacking in the literature, the criteria for transitioning to surgical evaluation are generally based on a combination of time and clinical milestones. Surgical referral is indicated when patients experience: (1) persistent ulnar-sided wrist pain that restricts activities of daily living or sports participation despite 3–6 months of structured rehabilitation; (2) failure to achieve clinically meaningful improvements in functional grip strength or active range of motion; or (3) the onset or persistence of clinical signs indicating DRUJ instability (35, 56, 57). Furthermore, the timing of surgical intervention should be tailored to the patient's functional demands; for high-demand individuals, such as elite athletes, early surgical referral may be considered at the 3-month mark if non-surgical management fails to restore requisite functional capacity (57).

### Structured rehabilitation framework

To address the heterogeneity of TFCC injuries and the lack of standardised non-surgical management protocols, this review proposes a structured three-phase rehabilitation framework for both acute traumatic and repetitive overuse-related TFCC injuries, adapted from prior work (58). The staging rationale is grounded in principles of tissue healing and functional motor learning (59, 60). Progression through each phase is guided by the achievement of predefined clinical objectives and the patient's functional recovery status, rather than rigid time-based criteria.

The framework advances progressively from Phase I, which focuses on symptom modulation and tissue protection, to Phase II, emphasising restoration of wrist range of motion and controlled tissue-loading exercises, and finally to Phase III, targeting neuromuscular control and functional reintegration. Notably, Phase I rehabilitation strategies are tailored to the underlying injury mechanism, with distinct approaches for acute traumatic versus repetitive overuse-related TFCC injuries. While Phases II and III share common intervention principles across injury types, the rate of progression through these phases must be individualised based on the underlying injury aetiology. For instance, an athlete recovering from an acute, structurally healed trauma may tolerate a more rapid progression through tissue loading and functional reintegration (61). Conversely, a patient managing a chronic, degenerative overuse injury typically requires a more prolonged and gradual period of load management to allow for adequate tissue adaptation and to prevent the exacerbation of insidious symptoms (62). Table 2 summarises a detailed, phase-specific non-surgical management pathway for stable TFCC injuries, integrating current evidence into a practical clinical framework.

**Table 2:** Stage-based rehabilitation exercises and objectives in TFCC non-surgical management.

Intervention Stage	Rehabilitation Objective	Management Strategies	Example Interventions	Clinical Reasoning	Progression Criteria
Phase I: Early Symptom Modulation—Acute trauma-related TFCC	Reduce pain and inflammation; protect injured tissue; maintain joint mobility	Orthosis or splint for wrist support (65)	Wear wrist splint or brace throughout the day	Protects healing tissue from excessive shear forces (65); reduces acute vascular response and edema; and inhibits inflammatory pathways pharmacologically (94)	Resting NPRS ≤3/10; ability to perform pain-free grip at 25% of the contralateral side; absence of night pain; tolerance of minimal active wrist movement without acute symptom exacerbation (87).
		Physiotherapy modalities	Cryotherapy (e.g., ice compresses) (67)		
		NSAIDs or corticosteroid injections (69)	Intra-articular corticosteroid injection to directly target local inflammation and provide short-term symptomatic relief		
Phase I: Early Symptom Modulation—Repetitive overuse-related TFCC	Reduce activity-related pain; prevent symptom aggravation; maintain functional movement	Short-term orthoses during high-load activities (71)	Use of a lightweight wrist sleeve or brace during high-load activities	Unloads the TFCC specifically during high-stress tasks to prevent flare-ups (71); empowers the patient to differentiate between adaptive tissue pain and injury-related damage (72).	NPRS ≤ 3/10 during baseline daily activities; ability to perform light functional tasks without delayed symptom flares (87).
		Evidence-based pain education (72)	Patient education on pain monitoring and load management		
Phase II: Progressive Tissue Loading	Promote tissue healing; restore joint mobility; re-establish neuromuscular control	Progressive muscular function training	Active range-of-motion and stretching exercises (Figure 4) (95)	Aims to restore accessory motion, modulate pain, and improve arthrokinematics glide; stimulates tissue remodelling via mechanotransduction (59).	Symptom stability during controlled loading; pain-free AROM ≥ 80% of the contralateral side; grip strength ≥ 70% of the contralateral side (85, 86); NPRS ≤ 3/10 during targeted resistance exercise.
			Joint mobilisation techniques (Figure 5) (76, 96)		
			Progressive strengthening exercises using low-load resistance (75, 97)		
		Adjunctive physiotherapy modalities	Thermotherapy (78), TENS (98), ultrasound (79), applied selectively for symptom modulation		
Phase III: Neuromuscular & Functional Reintegration	Restore functional wrist use; enhance neuromuscular control; integrate wrist function into daily and load-bearing tasks	Concentric and eccentric strengthening	Resistance band exercises performed in a neutral wrist position (Figure 6) (97)	Re-establishes proprioception and dynamic joint stability (58); restores efficient force transmission in sport- or occupation-specific movement planes (88).	NPRS ≤ 2/10 during advanced functional and weight-bearing tasks; bilateral wrist strength ≥ 85% of the contralateral side; full restoration of pain-free multi-joint coordination (85, 86).
		Neuromuscular control and stability training	Hand-supported Swiss ball weight-shift exercises against a wall (76)		
			Kettlebell stability holds performed with the wrist maintained in neutral alignment (91, 92)		
			PNF resistive coordination training		
		Functional task-oriented training	DTM-dart throwing motion (90)		
			Gradual reintroduction of task-specific activities requiring controlled wrist loading (e.g., reaching, lifting, supported weight-bearing)		

This structured pathway represents a conceptual framework proposed by the authors based on available literature and clinical reasoning, rather than a validated or prescriptive clinical guideline. It is intended to support clinical reasoning and promote a degree of standardisation, while recognising that all management decisions must be individualised. TFCC, Triangular fibrocartilage complex; AROM, Active Range of Motion; NPRS, Numerical Pain Rating Scale; NSAID, Non-Steroidal Anti-Inflammatory Drug; DWT, Dart Throwing Motion; PNF, Proprioceptive Neuromuscular Facilitation.

### Phase I: early symptom modulation—acute trauma-related TFCC presentation

This phase applies to patients with acute trauma–related TFCC injuries without DRUJ instability, who typically present with immediate ulnar-sided wrist pain, swelling, restricted motion, and reduced grip strength (63). The defining feature is the clinical presentation—namely pain and inflammation-driven dysfunction—rather than the injury mechanism itself. Management focuses on symptom modulation and protection of the injured structures from excessive mechanical stress, with primary goals to reduce inflammation, maintain joint mobility, and prepare the wrist for subsequent progressive loading and functional rehabilitation (64).

During the initial 0–2 weeks post-injury, wrist support with orthotics or splints is a key component of conservative management. Retrospective data suggest that bracing can increase relative load-bearing capacity of the injured wrist from 48% to 59% (65, 66). Concurrent activity modification and weight-bearing restriction further minimise mechanical stress and facilitate tissue healing. In addition, cryotherapy is recommended during the first 48 h, with ice packs applied for 20–30 min, three to four times daily (67, 68).

Pharmacologic interventions commonly include NSAIDs, such as celecoxib (200 mg/day) or meloxicam (7.5 mg/day), providing analgesic and anti-inflammatory effects. In cases of persistent pain, intra-articular corticosteroid injections may offer short-term relief (69). Notably, while NSAIDs have been shown to reduce pain over a six-week period, they do not significantly improve functional outcomes or address underlying structural pathology (70).

### Phase I: early symptom modulation—repetitive overuse-related TFCC presentation

This phase applies to patients with overuse-related TFCC injuries without DRUJ instability. Unlike acute trauma injuries, these develop gradually, with activity- and load-sensitive dysfunction rather than acute inflammatory symptoms. Early manifestations typically include dull, activity-related ulnar-sided wrist pain or soreness that is relieved by rest, which may progress to persistent discomfort during wrist motion. Management focuses on symptom modulation and protection of the involved structures through appropriate load management, while maintaining functional movement as tolerated. The primary goals are to reduce pain, prevent symptom aggravation, and establish a foundation for subsequent progressive loading and functional rehabilitation.

As discussed in the aetiology section, integrating a biopsychosocial approach—including patient education, reassurance, and addressing individual expectations—is an important component of management, particularly in overuse-related presentations.

Unlike acute trauma-related presentations, overuse-related TFCC injuries do not typically require prolonged rigid immobilisation. Evidence suggests that extended splinting may lead to discomfort and maladaptive loading, whereas short-term use of lightweight orthoses during high-load or symptom-provoking activities can provide adequate support while minimising adverse effects (71).

Evidence-based pain education is recommended to facilitate appropriate symptom interpretation and self-management. Rehabilitation should emphasise the distinction between injury-related pain and adaptive pain (72). Injury-related pain is characterised by persistent or activity-provoked symptoms that indicate excessive loading (72). In contrast, adaptive pain, such as mild transient soreness during early rehabilitation, reflects normal tissue adaptation rather than structural damage (72).

### Phase II: progressive tissue loading

With stabilisation of symptoms and restored baseline function, rehabilitation advances from tissue protection toward controlled loading and functional restoration. The primary aims are to optimise tissue healing, restore joint mobility, and re-establish neuromuscular control of the wrist and forearm.

Central to this phase is progressive muscular function training, which improves active range of motion (AROM), muscular coordination, and dynamic wrist stability (55). Structured, graded functional exercises introduced after symptom stabilisation enhance mobility, functional performance, and quality of life in patients recovering from TFCC injuries   (73, 74). Techniques such as dorsal glide mobilisation and low-intensity resistance training may further reduce pain and support functional recovery (75, 76).

Adjunctive physiotherapy modalities—including thermotherapy, ultrasound, transcutaneous electrical nerve stimulation (TENS), and acupuncture—can support symptom resolution and tissue recovery (77). Thermotherapy may enhance microcirculation and soft tissue extensibility, thereby improving AROM  (78). Ultrasound therapy shows potential for promoting tissue healing, though TFCC-specific evidence is limited and clinical judgement is required (79).

Emerging interventions such as extracorporeal shock wave therapy (ESWT), platelet-rich plasma (PRP), and electroacupuncture (EA) are under investigation for TFCC rehabilitation (80–84). Preliminary findings suggest benefits in pain reduction and tissue repair, but evidence is limited, heterogeneous, and largely extrapolated from other musculoskeletal conditions. These modalities should be considered investigational and applied selectively rather than as routine components of Phase II rehabilitation.

### Phase III: neuromuscular & functional re-integration

Phase III represents the final stage of non-surgical TFCC rehabilitation. It begins when resting pain is minimal (Numeric Pain Rating Scale [NPRS] ≤ 2/10), pain-free active range of motion (AROM) is at least 50%–75% of the asymptomatic contralateral side, and the wrist demonstrates tolerance to progressive loading without delayed symptom flares (85–87). The primary objectives are to restore functional wrist use in daily activities and achieve safe, pain-free self-weight-bearing capacity. Rehabilitation emphasises re-establishing neuromuscular control, intermuscular coordination, and integrated wrist function through graded, task-specific, and load-bearing activities (58). This phase marks the transition from isolated strengthening to coordinated, functional movement patterns, in accordance with principles of motor learning and tissue adaptation.

Evidence supports multi-directional wrist resistance training to improve torque control and muscular strength, which should be incorporated during advanced stages of progressive loading (75). Training progression should be guided by individual symptom response and functional performance rather than fixed time intervals. Dart Throwing Motion (DTM) training is also recommended to restore coordinated wrist motion within a functional plane (88). Biomechanical studies indicate that the wrist's mechanical axis and several muscle attachment sites align closely with the DTM plan (89, 90). This oblique plane, extending from radial extension to ulnar flexion, requires relatively low muscular effort and closely mirrors movement patterns used in daily life (88).

To further enhance wrist stability and grip strength, isometric exercises—such as kettlebell holds in a neutral wrist position—may be incorporated selectively (91, 92). These static exercises activate dynamic stabilisers while minimising shear forces across the TFCC (91). Isometric loading can be progressed by gradually increasing hold duration, introducing light external resistance (e.g., resistance bands or cable columns), or performing the exercises in more functional positions, such as controlled weight-bearing with the wrist maintained in a neutral alignment. Proprioceptive Neuromuscular Facilitation (PNF) techniques, particularly the diagonal D2 flexion–extension pattern, are employed to integrate wrist DTM within a proximal-to-distal kinetic chain, enhancing sensorimotor control and functional coordination (93).

As functional tolerance improves, rehabilitation can gradually progress to higher-demand daily and occupational tasks, with careful symptom monitoring to ensure sustained recovery without exacerbation.

### Limitations

This review has several limitations. As a narrative synthesis, it inherently lacks the methodological rigour and formal bias control of a systematic review, and selection and publication bias cannot be excluded. The non-surgical management pathway presented in Table 2 is derived from a synthesis of available literature; however, TFCC-specific rehabilitation data remain limited, heterogeneous, and inconsistently reported. While rehabilitation goals, therapeutic techniques, and progression criteria were informed by published studies, the overall staging framework was partially adapted from models used for other musculoskeletal conditions and supplemented by clinical experience. Consequently, the proposed pathway represents a conceptual framework based on available literature and clinical reasoning, rather than a validated, standardised, or prescriptive rehabilitation protocol. Overall, the sparse and methodologically weak evidence base—including physiotherapy modalities, extracorporeal shockwave therapy, platelet-rich plasma, and electrotherapies—restricts the ability to provide standardised, TFCC-specific treatment prescriptions. Therefore, this framework should be applied cautiously and adapted to individual patient characteristics, injury patterns, and functional demands. Future high-quality, TFCC-specific studies with standardised rehabilitation reporting are required to strengthen the clinical applicability of conservative management recommendations.

## Conclusions

Current evidence supports non-surgical management as the first-line approach for clinically stable TFCC injuries with an intact DRUJ, while surgical intervention is reserved for unstable patterns. To address differences in injury mechanisms, particularly between acute trauma-related and repetitive overuse-related degenerative presentations, a structured, phase-based rehabilitation framework is proposed for stable injuries. This framework is derived from available literature and clinical reasoning to support clinical decision-making and standardise care. Emerging therapies such as ESWT and PRP remain investigational due to limited TFCC-specific evidence. Overall, these insights provide a consolidated roadmap for non-surgical TFCC management and highlight the need for high-quality studies to validate rehabilitation protocols and novel interventions.
